# Investment strategies for sustainable safe development of Chinese coal mine employees driven by digital intelligence

**DOI:** 10.3389/fpubh.2024.1464930

**Published:** 2024-12-13

**Authors:** Yuan Yuan, Gang Cheng, Weicai Peng, Xia Yang, Yamin Du

**Affiliations:** ^1^School of Economics and Management, Huainan Normal University, Huainan, China; ^2^School of Engineering Sciences, University of Science and Technology of China, Hefei, China; ^3^School of Mathematics and Big Data, Chaohu University, Chaohu, China

**Keywords:** digital intelligence, surveillance, team, management, investment strategy, sustainable development

## Abstract

China’s “14th Five-Year Plan” proposes the construction of a “Digital China,” posing the challenge of digital transformation to coal mining enterprises. It is critical to compare the effectiveness of investing in digital devices with that of human capital. This study establishes a structural equation model based on the ‘regulation-situation-behavior’ theoretical framework. The model, developed through in-depth empirical analysis of enterprises, captures the relationships between exogenous and endogenous latent variables. The primary factors influencing both the active and passive safety behaviors of coal miners are discussed. The micro-mechanisms of human interaction with digital intelligence equipment are analyzed. The findings indicate that, in terms of overall utility value, investment in Intelligent surveillance management generates a total utility value that is 4.292 times higher than that of investment in team demonstration management. This disparity is primarily attributed to the significant positive impact that Intelligent surveillance management exerts on the active safety behavior of coal miners. Specifically, it influences miners’ safety behavior through the dual effects of situational promotion focus and situational prevention focus, whereas team demonstration management solely utilizes situational promotion focus. Additionally, the investigation reveals that miners attach significant importance to the role of instant feedback and continuous monitoring in Intelligent surveillance management. Consequently, coal mining enterprises should prioritize investing in digital intelligence supervision systems with real-time, full-time, and full-coverage capabilities. They should also focus on improving education, publicity, and training related to Intelligent surveillance management. These approaches can effectively enhance the digital, intelligent, safe and sustainable development capabilities of coal mines.

## 1 Introduction

Globally, China boasts a significant role in the energy sector as a pivotal energy producer and consumer. As stated in the “China Statistical Yearbook (2023),” In 2022, the country’s raw coal production accounted for 67.4% of the total primary energy production, while coal consumption constituted 56.2% of the total energy consumption (1). It can be deduced that coal holds a subject status in energy mix.

Chinese Mineral Resources Report (2023) shows that since 2022, the fixed assets investment in mining industry has continued to grow, and the annual growth rate of fixed assets investment in coal mining and washing industry is 24.4% higher than the prior year (2). With the strengthening of coal as a guaranteed energy source, the investment in coal mining has shown a continuous increase trend.

However, the sustainability of the coal industry faces serious challenges. Firstly, some coal mining regions are nearing the end of their mining lifespan, while most are transitioning to deep mining, which significantly increases the frequency of hazardous accidents (3). Secondly, any risk accident resulting in casualties prompts rigorous inspections, rectifications, or even mine closures, rendering operations unsustainable (4). Finally, the coal mining industry, a notoriously arduous and hazardous sector, suffers from a significant loss of young, highly educated workers, thereby impeding the sustainable development of mine human capital (5). As a result, the pressure on coal mines’ sustainability has significantly escalated the uncertainty and instability of mineral investments.

The development of digital intelligence technologies, such as big data and artificial intelligence, has ushered in novel opportunities for the sustainable development of coal mines (6). These technologies enhance the level of coal mine safety management and mitigate accident risks through real-time monitoring, intelligent warning systems, data analysis, and other methods (7). Simultaneously, digital intelligence technology aids investors in gaining a deeper understanding of coal mine production conditions and market dynamics, thereby providing more accurate and timely information to support investment decisions (8). While numerous researchers have actively examined the application of digital intelligence technology in coal mines, focusing primarily on its role in boosting production efficiency and mitigating safety risks, there is a paucity of research analyzing the impact of human capital and digital intelligence equipment on the sustainable development of coal mines. Specifically, the differences in safety effectiveness between investing in human capital and digital intelligence equipment have received limited attention. Moreover, with the rapid advancement of generative artificial intelligence technology, particularly in labor-intensive coal mining enterprises, where human capital levels have stagnated over the past decade, this comparative study holds significant research importance and practical value. Researchers alike urgently require guidance to assess the relative effectiveness of investing in digital intelligence equipment versus human capital.

It is noteworthy that digital intelligence equipment primarily impacts safety and sustainability by influencing human behavior, specifically the actions of individuals (9). Seventy percent of coal mine safety accidents stem from unsafe production practices involving human operators (10–14). Researchers require an entry point that reaches the analysis of human unsafe behavior and its underlying motivations. The situational focus theory offers a theoretical framework to comprehend the moderating mechanism in a specific work context (15). In the domain of coal mine worker safety, digital intelligence monitoring, as an emerging management approach, aligns closely with the situational focus theory. Managers can utilize this theory to systematically explore the application of intelligent surveillance in coal mine work scenarios and formulate more precise and effective investment strategies grounded in the behavioral characteristics and psychological mechanisms. By optimizing resource allocation, enhancing employees’ safety awareness, and strengthening safety management, the sustainable development of coal mine safety can be achieved. This approach also provides theoretical support and practical guidance for scientific and technological innovations in coal mine safety management.

## 2 Literature review

Chinese coal mine digital intelligence transformation market holds immense potential, with the total investment scale anticipated to surpass 200 billion yuan (16). This highlights the critical nature of researching investment strategies for the digital intelligence of coal mines, as it can optimize resource allocation and foster sustainable development within the industry.

Globally, there is sustained enthusiasm for cutting-edge research in digital intelligence technology, innovative security management investigations, and global trend studies on sustainable development, leading to significant academic achievements in these respective fields (17–20). Among them, the research on the sustainable development of mines driven by digital intelligence is primarily focused on green mining technology, intelligent mine construction, and circular economy. Specifically, regarding the research on the driving role of digital intelligence technology in mine safety development, the current focus is on intelligent mining, monitoring, and early warning. However, there is a lack of relevant research on the human-machine coupling effect driven by digital intelligence. As for the human-machine coupling, the safety behavior of coal miners has always been the core content. Internationally, the research on coal miners’ safety behavior primarily comprises studies on individual behavior and group behavior. The school of individual behavior research mainly focuses on the impact of factors such as workers’ psychology, physiology, skills, and experience on safety behavior (21). The proposition suggests that safety attitude is a crucial factor affecting the safety behavior of coal miners (22, 23). Research at the group level focuses on the impact of factors such as interaction, communication, and organizational culture among workers on safety behavior (24, 25). For instance, effective team demonstration management can effectively reduce the occurrence of safety accidents (26–28). The Situational Focus Theory (SFT) does not distinguish between groups and individuals. It comprises situational promotion focus and situational prevention focus (29). The situational promotion focus mainly concerns whether workers’ behaviors will lead to positive outcomes (30), while the situational prevention focus concerns on whether their behaviors will result in negative outcomes (31). The SFT emphasizes the importance of contextual factors in miners’ behavioral decisions (32, 33), providing a new perspective for understanding and improving the safety behavior (32, 34, 35). It also offers a new analytical tool for exploring potential behavioral trends among coal miners in the new digitalized underground work environment (36).

Production managers in coal mines believe that team management is the frontline grass-roots management unit closest to miners’ safe production behavior. Under the condition of digitalized production, compared with the influence of team demonstration management on safety behavior, the relative impact of Intelligent surveillance management on the safety behavior of frontline coal miners urgently needs to be verified. However, the utility relationship between coal miners’ safety behaviors, especially proactive and reactive safety behaviors, and intelligent surveillance management is unclear. Foreign psychologists suggest that workers have two modes of attempting to achieve their goals: prevention focus and promotion focus (37). Given that team demonstration management and intelligent surveillance management constitute the work environment directly encountered by modern coal miners, it is scientifically valuable to investigate the effects of exogenous latent variables such as team demonstration management and intelligent surveillance management on endogenous latent variables, namely proactive and reactive safety behaviors, with situational promotion focus and situational prevention focus as mediating variables. This exploration can help analyze the utility ratio between investing in digitalized monitoring equipment and investing in high-quality human capital, providing valuable support for scientific decision-making.

Thus, to comply with the government’s policy requirements for the application of intelligent technology in coal mines, enhance the sustainability of enterprises, and achieve better investment utility, as well as to improve the theory and practice of human-machine coupling driven by digital intelligence in coal mining enterprises, this study analyzes the advantages and disadvantages of digitalized surveillance management and traditional team demonstration management in coal mine safety production, and explores the human-machine coupling mechanism in digital intelligence-driven safety management. The research not only expands and deepens the situational focus theory from a theoretical perspective but also applies it to the latest digitalized development of the Chinese coal mining industry, verifying and enriching the theory’s applicability and explanatory power in specific cultural and industry contexts. The study emphasizes the crucial role of digital intelligence technology in promoting the sustainable development of miner’ safety, providing theoretical support and practical guidance for safety investment decisions in the coal industry. At the practical level, the findings can help guide coal enterprises in how to utilize digital intelligence technology to optimize safety production, while also providing important references for the formulation of effective safety investment strategies.

## 3 Model construction

**Figure 1:**
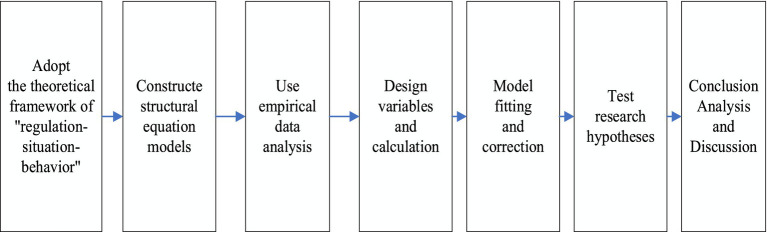
The research flow chart.

To delve deeply into how digital intelligence technology drives investment strategies for the safety and sustainable development in Chinese coal mines, the research team conducted an investigation in production units of coal mines. Such as questionnaire surveys and in-depth interviews were employed to carefully quantify and compare the effectiveness of modern management techniques (Intelligent surveillance management) and traditional management methods (Team demonstration management) in enhancing the safety behaviors of coal miners. By combining the specific modulating effects of work contexts, this study aims to reveal the functional paths of Intelligent surveillance management under different scenarios and quantify its impact on improving coal miners’ safety awareness and behavioral norms. The overall research technical route is shown in Figure 1.

### 3.1 Build a research model and design variables

Structural equation modeling (SEM) is a descriptive representation of the relationship between latent variables. It can be used to represent the relationships among several variables and also to examine the relationships between groups of variables. Essentially, the relationships between observable group variables reflect the relationships among latent variables. SEM comprises two subclasses: measurement models and structural models, corresponding to two types of equations: measurement equations and structural equations. Measurement equations can be expressed as:

The measurement models for Intelligent surveillance management of external latent variables *X_1_* and team demonstration management *X_2_* are shown in Equations 1 and 2 as follows:


(1) $$X_{1}=\gamma_{X1}\xi_{1}+\delta_{X1}$$

(2) $$X_{2}=\gamma_{X2}\xi_{2}+\delta_{X2}$$

The measurement models for the endogenous latent variables of proactive safety behavior *Y_1_* and passive safety behavior *Y_2_* among coal miners are shown in Equations 3 and 4.


(3) $$Y_{1}=\gamma_{Y1}\;\eta_{1}+\varepsilon_{Y1}$$

(4) $$Y_{2}=\gamma_{Y2}\;\eta_{2}+\varepsilon_{Y2}$$

The measurement models for the mediating variables of situational promotion focus effect *Z_1_* and situational prevention focus effect *Z_2_* are shown in Equations 5 and 6 as follows:


(5) $$Z_{1}=\gamma_{z1}\zeta_{1}+\delta_{z1}$$

(6) $$Z_{2}=\gamma_{z2}\zeta_{2}+\delta_{z2}$$

Structural models can be constructed to explain the relationships between latent variables:

Among them, the influence of exogenous latent variables on mediator variables are shown in Equations 7 and 8.


(7) $$\zeta_{1}=\omega_{11}\xi_{1}+\omega_{12}\xi_{2}+\zeta_{1res}$$

(8) $$\zeta_{2}=\omega_{21}\xi_{1}+\omega_{22}\xi_{2}+\zeta_{2res}$$

The influence of mediator variables on endogenous latent variables are shown in Equations 9 and 10.


(9) $$\eta_{1}=\alpha_{11}\zeta_{1}+\alpha_{12}\zeta_{2}+\eta_{1res}$$

(10) $$\eta_{2}=\alpha_{21}\zeta_{1}+\alpha_{22}\zeta_{2}+\eta_{2res}$$

**Figure 2:**
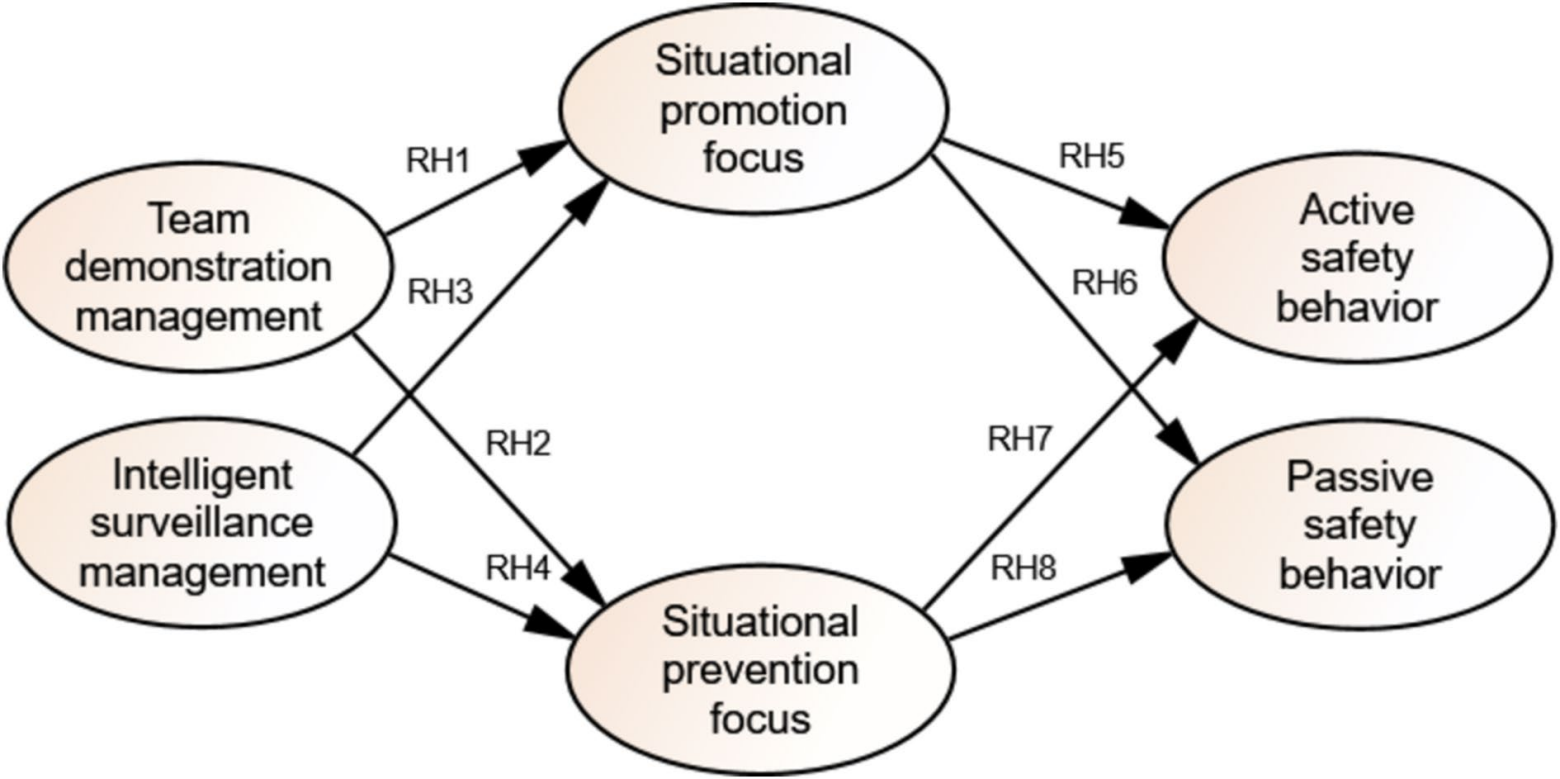
Research model of miners’ safety behavior.

*γ* represents the factor loading, *ω* and *α* represent the path coefficients, while *δ*, *ζ_res_*, and *η_res_* represent measurement errors and residuals, respectively. The obtained structural equation model is shown in Figure 2.

According to the research relationship, the measurement model is constructed in Figure 2, and the research hypotheses are established as follows:

RH1: Team demonstration management exerts a significant positive influence on situational promotion focus.

RH2: Team demonstration management has a notable positive effect on situational prevention focus.

RH3: Intelligent surveillance management significantly and positively impacts contextual promotion focus.

RH4: Intelligent surveillance management has a significant positive influence on situational prevention focus.

RH5: Situational promotion focus positively affects coal miners’ active safety behavior.

RH6: Situational promotion focus significantly and positively impacts the passive safety behavior of coal miners.

RH7: Situational prevention focus has a notable positive effect on coal miners’ active safety behavior.

RH8: Situational prevention focus significantly and positively influences the passive safety behavior of coal miners.

For the design of research variables, in the analysis of intelligent surveillance management of latent variables, a large number of existing literatures have not found mature measurement scales about intelligent surveillance management to miners’ safety behavior, for that reason, on the basis of using the Miners’ Unsafe Behavior Management Measurement Scale (38), the research team considered the actual utility of “intelligent surveillance management” as an external potential variable, *EIMP1*, *EIMP2* and *EIMP3* in the scale are listed as dominant variables based on “situational prevention focus,” and *EIMF1*, *EIMF2*, *EIMF3*, *EIMF4* and *EIMF5* in the scale are listed as dominant variables based on “situational promotion focus.” As an innovative scale, this subscale employs both exploratory and confirmatory analysis for analysis.

In the analysis of Team Demonstration Management as a latent variable, the effect scale of team leader leadership style is adopted (39). Reference is made on appointing grass-roots supervisors as work-life role models (40), Utilizing “Team Demonstration Management” as the latent variable, *GRM1*, *GRM2*, and *GRM3* are designated as observable variables.

In the ongoing research of situational focus theory, the situational focus scale remains a significant topic of interest in psychology and behavior (41).Currently, this scale has reached a high level of maturity. This study combines exploratory research on Regulation Focus Questionnaire (RFQ) based on Chinese work situation and Wallace’s Regulation Focus at Work Scale (RWS) based on specific safe work situation, the scale contains 12 items, where *FSP1* to *FSP6* assess the effect of situational prevention focus, while *FSF1* to *FSF6* evaluate the impact of situational promotion focus.

The safety behavior scale has reached a state of maturity. Drawing upon the research findings (31, 42, 43), the research team has distilled the miners’ safety behavior into two distinct dimensions: Active safety behavior and passive safety behavior.

The detailed composition of the scale, along with a concise description of the relevant measurement items, is presented in Table 1.

**Table 1:** Reliability analysis results of sample data.

Latent variable	Brief description of related measurement items	Observation variable	CITC	Cronbach’s *α*	Reliability evaluation
Intelligent surveillance management	Real-time risk monitoring	*EIMP1*	0.721	0.917	Very high reliability
	Full-time risk prediction	*EIMP2*	0.762		
	Risk decision support	*EIMP3*	0.729		
	Early warning of potential risks	*EIMF1*	0.790		
	Standardized operation monitoring	*EIMF2*	0.716		
	Emergency assistance management	*EIMF3*	0.678		
	Duty compliance monitoring	*EIMF4*	0.724		
	Facilitate the establishment of safety awareness and the development of safety skills	*EIMF5*	0.697		
Team demonstration management	Recognize the team safety model	*GRM1*	0.712	0.839	High reliability
	Approve the safety regulations for teams	*GRM2*	0.716		
	Recognize the safety demonstration of the team	*GRM3*	0.682		
Situational promotion focus	Desire to complete the work	*FSF1*	0.680	0.904	Very high reliability
	The determination to finish the task	*FSF2*	0.761		
	Emphasize the efficiency of work	*FSF3*	0.765		
	Emphasize the precision of work	*FSF4*	0.757		
	Emphasize the sense of accomplishment in work	*FSF5*	0.765		
	Focus on the quantity of tasks	*FSF6*	0.691		
Situational prevention focus	Prevent work violations	*FSP1*	0.622	0.816	High reliability
	Prevent work timeouts	*FSP2*	0.699		
	Prevent work errors	*FSP3*	0.721		
	Prevent low-quality completions	*FSP4*	0.679		
	Prevent inefficient completions	*FSP6*	0.381		
Active safety behavior	Proactive compliance with safety regulations	*ASB1*	0.734	0.921	Very high reliability
	Take the initiative to participate in safety training	*ASB2*	0.741		
	Actively participate in safety production	*ASB3*	0.742		
	Proactively report on safety work	*ASB4*	0.800		
	Actively seek safe methods	*ASB5*	0.777		
	Actively enhance the security environment	*ASB6*	0.753		
	Actively improve safety efficiency	*ASB7*	0.729		
Passive safety behavior	Passive compliance with safety regulations	*PSB1*	0.503	0.926	Very high reliability
	Passive wearing of protective equipment	*PSB2*	0.869		
	Passive participation in safety training	*PSB3*	0.913		
	Passive participation in safety education	*PSB4*	0.893		
	Passive participation in safety assessment	*PSB5*	0.861		

### 3.2 Sample collection and data collation

The Huaibei-Huainan region in China, situated in the economically significant area of East China, exhibits a relatively high level of intelligent development in coal mines. Questionnaires were dispensed among front-line production unit employees of Huaihei Energy Group, Huaibei Coal and Electricity Group, Wanbei Coal and Electricity Group, and China National Coal Group Xinji in the designated region. A total of 360 questionnaires were issued, resulting in the retrieval of 317 completed forms. Subsequently, upon careful scrutiny, questionnaires containing obvious errors or incomplete information were discarded, yielding a final tally of 296 valid questionnaires. This translated to an effective questionnaire recovery rate of 82.22%. The Likert 5 method was employed for the measurement of the questionnaire. The outcomes of the demographic variable analysis of the sample population are detailed in Table 2.

**Table 2:** Statistical analysis table of sample population.

Sex composition and proportion of samples
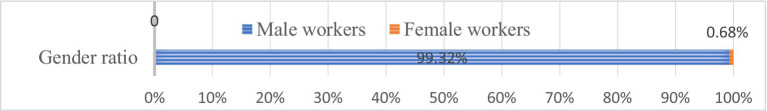
Age composition and proportion of samples
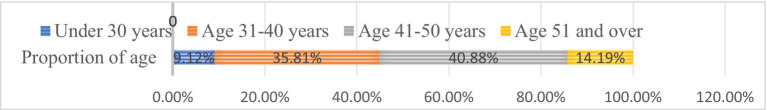
Composition and proportion of job types in the samples
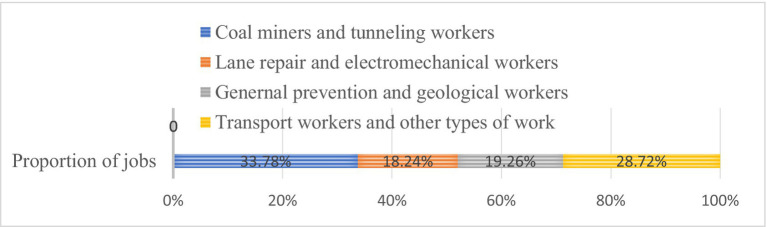
The composition and proportion of the length of service of the samples
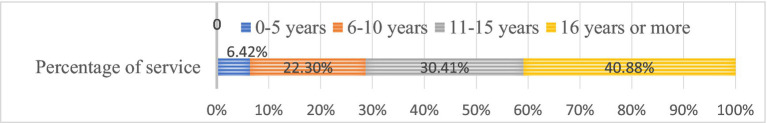
The composition and proportion of education level of samples
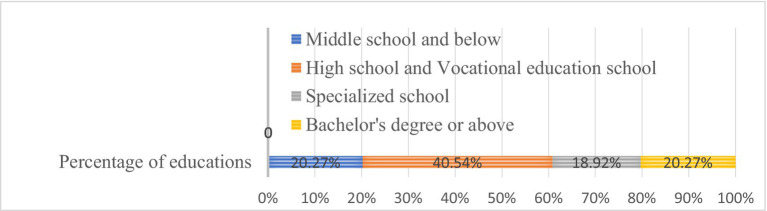

From the statistical analysis results of the sample population, It is evident that male miners constitute the majority in the gender ratio, aligning with the characteristic gender distribution among front-line workers in this industry; In terms of age distribution, miners are primarily concentrated in the middle-aged bracket of 41 to 50 years old, indicating an older average age among front-line coal mine workers. Regarding job roles, coal miners and tunneling workers constitute the majority of grass-roots miners, accounting for 33.78%. These two occupations pose the highest risks in front-line production, characterized by hazardous and volatile working conditions, demanding high levels of professional skills and knowledge literacy from employees. However, in terms of seniority, the proportion of coal miners with extensive vocational skills (with 15 years or more of service) stands at 40.88%, while those with less than 10 years of service make up 28.72%. Notably, due to the significant mobility of miners, the statistical service duration represents total working hours rather than those spent in a fixed unit. Additionally, when examining the educational background of the sample, it becomes evident that only 20.27% of coal workers hold higher education qualifications. The primary educational group comprises 40.54% with high school and vocational education, while another 20.27% possess junior high school education or below.

The educational level of team staff significantly impacts the demonstration safety management of traditional grass-roots teams in coal mines, generally speaking, employees’ safety understanding ability, risk identification and coping ability, learning and innovation ability, communication and writing ability, as well as safety awareness and attitude, are positively associated with their educational background. Consequently, front-line employees tend to be predominantly male, older in age, have average work experience, low educational backgrounds, and overall, exhibit substandard overall quality.

### 3.3 Reliability and validity analysis of sample data

In order to assess the data reliability of the questionnaire, the “reliability analysis” function of SPSS27 was utilized to examine the internal consistency of the questionnaire test.

Generally, when it comes to intelligent surveillance management and team demonstration management, the Krumbach coefficient typically exceeds 0.9, indicating that the questionnaire exhibits excellent internal consistency. A Crownbach coefficient above 0.8 suggests a high level of reliability for the questionnaire. However, if the Crownbach coefficient falls below the critical threshold of 0.7, it indicates that the questionnaire’s reliability is inadequate and requires adjustment.

During the initial round of reliability testing, it was discovered that all latent variables, except for the situational prevention focus, exhibited Crownbach coefficients higher than 0.8. This suggests that the original questionnaire was highly reliable, with the exception of the situational prevention focus. Upon analyzing the observed variables within the focus of situational prevention, it was found that variables *FSP4* and *FSP5* exhibited multicollinearity. Consequently, variable *FSP5* was eliminated, and a second round of reliability testing was conducted. The results of this testing, as presented in Table 1, demonstrate that all variables within the measurement model now possess high reliability.

In conducting a validity analysis of the questionnaire data, it is generally accepted that a KMO (Kaiser-Meyer-Olkin) value above 0.7 and a significant Bartlett’s test of sphericity indicate that the questionnaire data is appropriate for factor analysis. As demonstrated in Table 3, the measurement model has a KMO value of 0.940 and a Sig. value of 0.000, which strongly suggests that the test data is highly suitable for factor analysis.

**Table 3:** KMO and Bartlett test results of sample data.

Quantity of KMO sampling suitability		0.940
Bartlett sphericity test	Approximate chi-square	7890.352
	Degree of freedom	561
	Significance	0.000

### 3.4 Exploratory factor analysis

Using the factor analysis function of SPSS27, the data of 34 items were analyzed through exploratory factor analysis. The results are shown in Table 4.

**Table 4:** Explanation of common factor variance.

Composition	Extract the sum of squares of loads			Sum of squares of rotational loads		
	Total	Percentage	Accumulation %	Total	Percentage	Accumulation %
1	14.302	42.064	42.064	5.789	17.028	17.028
2	3.752	11.036	53.099	4.382	12.888	29.916
3	2.036	5.989	59.088	4.045	11.897	41.813
4	1.557	4.579	63.667	4.029	11.851	53.664
5	1.157	3.403	67.070	3.012	8.859	62.523
6	1.083	3.185	70.255	2.629	7.732	70.255

The extraction method was “principal component analysis” and the rotation method was “Caesar normalized maximum variance method.” The rotated matrix components show that 6 common factors are extracted from 34 measurement indexes of the survey sample data, and the cumulative interpretation variance is 70.255%.

### 3.5 Hypothetical model and confirmatory factor analysis

In order to further verify the results of exploratory factor analysis, on the one hand, to test whether the factor load of each observed variable is significant in the parameter estimation, on the other hand, to test the reliability and validity of each potential variable in the structural model.

**Figure 3:**
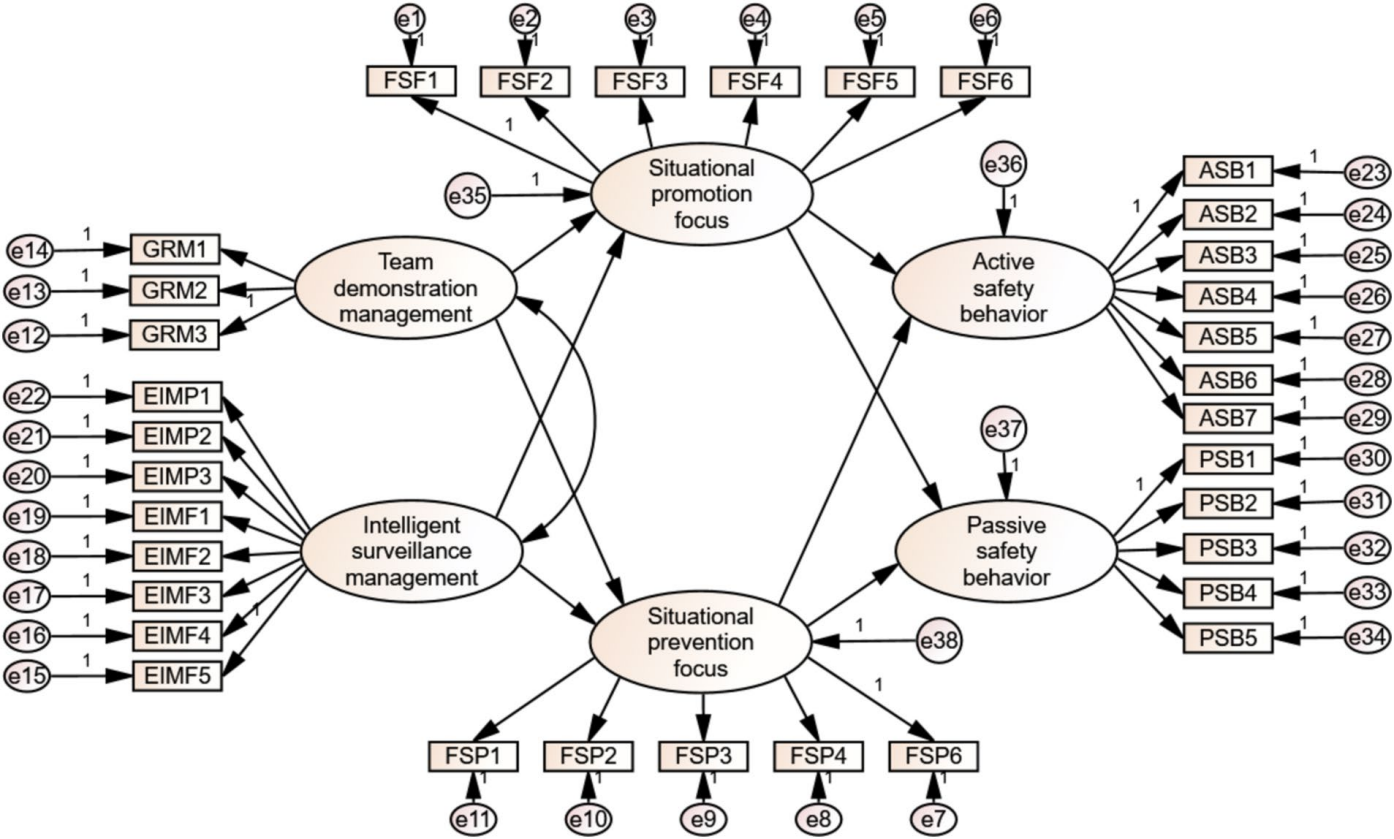
Index and evaluation of overall model adaptation degree of initial model.

The model underwent Confirmatory Factor Analysis (CFA) using AMOS24. The initial hypothetical model for confirmatory factor analysis is shown in Figure 3. The fitness test of the model examines the degree of agreement between the initial hypothetical model and the actual questionnaire data pointing to the assessment objectives. According to Wu Minglong’s suggestion (44), the model fitness indicators to be reported include absolute fitness indicators and relative fitness indicators, and the model test fitness indicators are shown in Table 5.

**Table 5:** Initial mode adaptability test values.

Test index	Absolute fitness index			Relative fitness index			Reduced fitness index		
	CMIN/DF	RMSEA	GFI	NFI	IFI	CFI	PNFI	PCFI	PGFI
Parameter value	2.736	0.077	0.771	0.828	0.883	0.883	0.764	0.815	0.672
Adaptability	Good	Good	Poor	General	General	General	Good	Good	Good

Other parameters: Number of samples = 296; CMIN = 1417.372; *p* = 0.000; DF = 518.

It can be seen from Table 5 that the fitness index of most models meets the model fitting standard, but the GFI value is 0.771, which does not meet the acceptable standard of model fitting, so the models must be revised.

### 3.6 Model modification and fitness test

**Figure 4:**
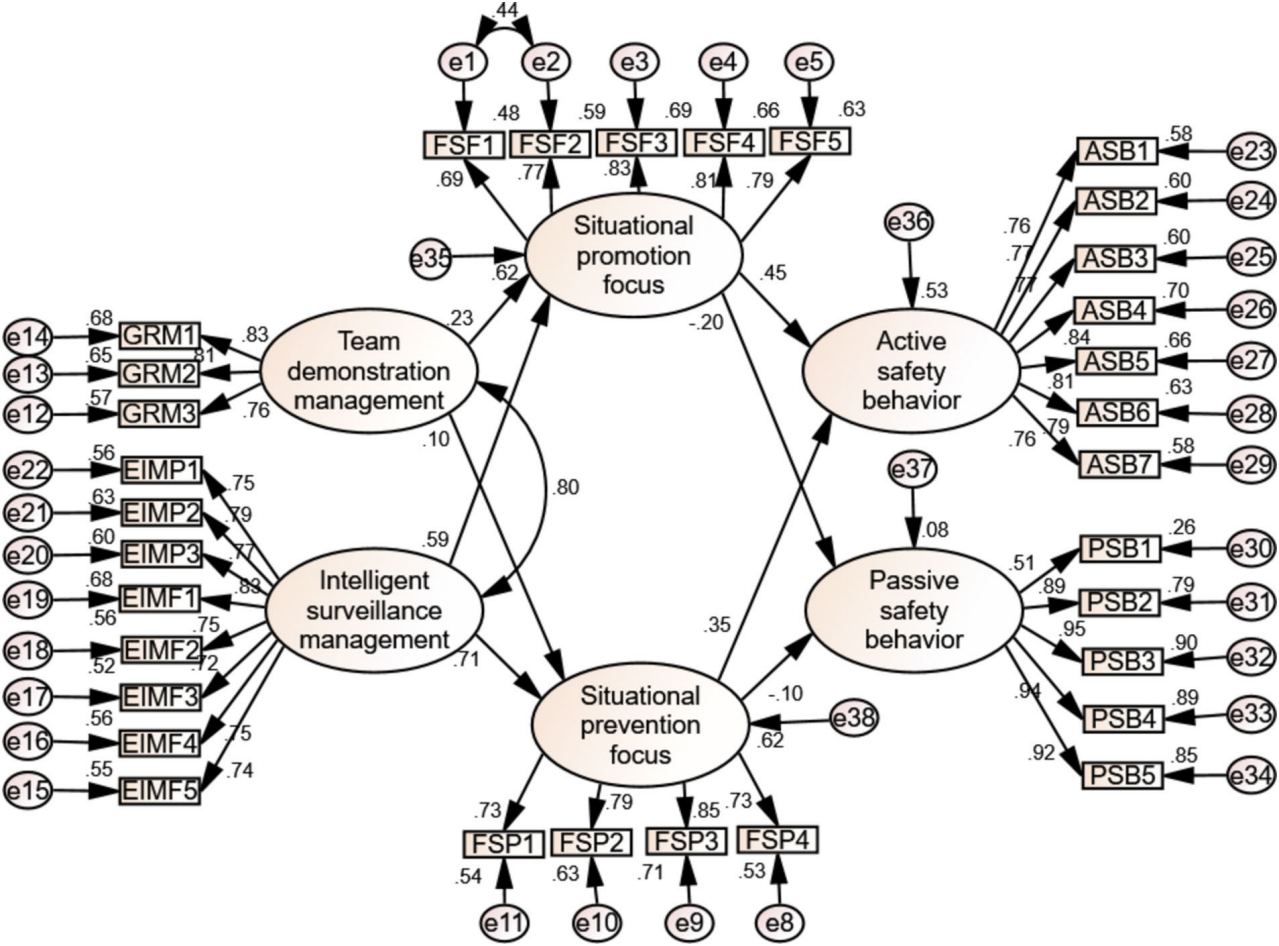
Adaptability index and evaluation of modified model.

The correction is based on the Modification Indices of the AMOS software. Corrections must have practical theoretical significance and correction value. The operation of the model after correction in accordance with the principle of correction and the results of the analysis of modification indices is shown in Figure 4.

The item-by-item analysis indicates that *FSF6*, “Focus on the quantity of tasks,” and *FSF3*, “Emphasize the efficiency of work,” exhibit partial overlap when analyzed within the context of Situational promotion focus, specifically around the theme of “people actively pursuing values.” When exploring the theme of “people actively pursuing value and work efficiency” within this focus, a partial overlap is observed, with the residual item e6 displaying a correlation chi-square value of 177.91, which is the highest sum of single-item correlation MI values recorded in the initial test. Furthermore, in the analysis of Situational prevention focus, *FSP6*, “Prevent inefficient completions,” was found to overlap partially with *FSP2*, “Prevent work timeouts.” Additionally, *FSP3*, “Prevent work errors,” demonstrated partial overlap when analyzed within the context of situational prevention. Notably, the residual term *e7* exhibited a correlation chi-square value of 129.45, ranking as the second highest sum of MI values for a single correlation in the first test. The analysis of the single correlation MI index reveals that establishing a correlation between the residual term *e1* and the residual term *e2* results in a reduction of the chi-square value by 52.39.

After the operation, the residual error of the modified model remains positive and significant. Additionally, the average normalized load coefficient exceeds 0.7, while the non-normalized load coefficient is also significant. Consequently, the running result of the model is excellent.

As shown in Table 6, the overall adaptability index of the revised model has significantly improved when compared to the revised index, indicating that the overall adaptability of the revised model is in a satisfactory state.

**Table 6:** Test values of fitness degree of modified model.

Test index	Absolute fitness index			Relative fitness index			Reduced fitness index		
	CMIN/DF	RMSEA	GFI	NFI	IFI	CFI	PNFI	PCFI	PGFI
Parameter value	2.432	0.070	0.808	0.857	0.911	0.910	0.784	0.833	0.695
Adapt-ability	Good	Good	General	General	Good	Good	Good	Good	Good

Other parameters: Number of samples = 296; CMIN = 1417.372; *p* = 0.000; DF = 518.

Furthermore, the test results obtained from running the revised model have been comprehensively organized, and the degree of support for each research hypothesis is clearly presented in Table 7.

**Table 7:** Coefficient test results of modified model.

Path relationship between latent variables	Non-normalized path coefficient	Standardized path coefficient	S.E.	C.R	*p*	Inspection result
Intelligent surveillance management→Situational promotion focus	0.509	0.454	0.090	5.664	***	Significant
Intelligent surveillance management→Situational prevention focus	0.684	0.707	0.105	6.531	***	Significant
Team demonstration management→Situational promotion focus	0.219	0.233	0.093	2.368	0.018	Significant
Team demonstration management→Situational prevention focus	0.103	0.098	0.104	0.992	0.321	Not significant
Situational promotion focus→Active safety behavior	0.483	0.454	0.088	5.459	***	Significant
Situational promotion focus→Passive safety behavior	−0.221	−0.203	0.102	−2.161	0.031	Significant
Situational prevention focus→Active safety behavior	0.336	0.352	0.077	4.391	***	Significant
Situational prevention focus→Passive safety behavior	−0.098	−0.100	0.090	−1.092	0.275	Not significant

S.E. refers to the standard error value; C.R. refers to the critical ratio; *p* was significant, ***means *p* < 0.001, Generally speaking, when the double-tail test *p* < 0.05, it means that the path coefficient estimation is significantly different from 0, It means that the normalized path coefficient is significant.

As can be seen from Table 7, except for the coefficient test of “team demonstration management→situational prevention focus” and “situational prevention focus→ passive safety behavior,” the other path coefficient results are significant. In evaluating the direct utility of intelligent surveillance systems, intelligent surveillance management has a significant positive effect on the focus of situational adjustment, among them, the standardized path coefficient of intelligent surveillance management to the focus of situational prevention is 0.707; the standardized path coefficient of Intelligent surveillance management to scene promotion focus is 0.454.

When comparing team demonstration management, the path coefficient for “team demonstration management→situational promotion focus” is significant, but its standardized path coefficient is 0.233. Conversely, the path coefficient for “team demonstration management→situational prevention focus” is not significant. Hence, it is evident that traditional team demonstration management is less effective than modern intelligent surveillance management in positively influencing the focus of situational adjustment.

Regarding the direct effect of the contextual adjustment focus, “situational promotion focus→active safety behavior” has a significant positive impact with a standardized path coefficient of 0.454. Similarly, “situational prevention focus→ active safety behavior” also has a significant positive effect, with a standardized path coefficient of 0.352. In terms of safety behavior, both situational promotion focus and situational prevention focus have a moderating effect, with the moderating effect of situational promotion focus being greater than that of situational prevention focus.

## 4 Results

### 4.1 Research hypothesis test results

Based on the content of the research hypotheses and the results of the structural equation analysis, the outcomes of the hypothesis testing are presented in Table 8. According to the analysis, the significance test results for RH2 and RH8 are not significant, therefore, we reject the hypotheses RH2 and RH8. Furthermore, the presumed positive effect of *RH6* does not align with the practical test results. Specifically, the test outcomes indicate that the situational promotion focus has a significant negative impact on the passive safety behavior of coal miners. Consequently, RH6 is rejected due to the inconsistency between its presumed positive effect and the actual test results.

**Table 8:** Research hypothesis test results.

Original hypothesis test	Path coefficient	Significance test	Hypothesis test results
RH1: Team demonstration management has a significant positive impact on situational promotion focus	0.233	Significant	Establish
RH2: Team demonstration management has a significant positive impact on situational prevention focus	0.098	Not significant	Fail
RH3:Intelligent surveillance management has a significant positive impact on situational promotion focus	0.454	Significant	Establish
RH4: Intelligent surveillance management has a significant positive impact on situational prevention focus	0.707	Significant	Establish
RH5: Situational promotion focus has a significant positive impact on active safety behavior	0.454	Significant	Establish
RH6:Situational promotion focus has a significant positive impact on passive safety behavior	−0.203	Significant	Fail
RH7:Situational prevention focus has a significant positive impact on active safety behavior	0.352	Significant	Establish
RH8: Situational prevention focus has a significant positive impact on passive safety behavior	−0.100	Not significant	Fail

The path coefficients of RH1, RH3, RH4, RH5, and RH7 are all greater than 0, meeting the significance requirements at various significance levels. The results of the hypothesis tests indicate the following: RH1: Assuming that team demonstration management has a significant positive impact on situational promotion focus is valid.

RH3: Assuming that intelligent surveillance management has a significant positive impact on situational promotion focus holds true.RH4: Assuming that intelligent surveillance management has a significant positive impact on situational prevention focus is accurate.RH5: Assuming that situational promotion focus has a significant positive impact on active safety behavior is correct.RH7: Assuming that situational prevention focus has a significant positive impact on active safety behavior is justified. Path analysis results demonstrate that intelligent surveillance management can influence miners’ active safety behavior through two mediating variables: situational promotion focus and situational prevention focus. Conversely, team demonstration management can solely affect miners’ active safety behavior through one mediating variable, namely situational promotion focus.

**Figure 5:**
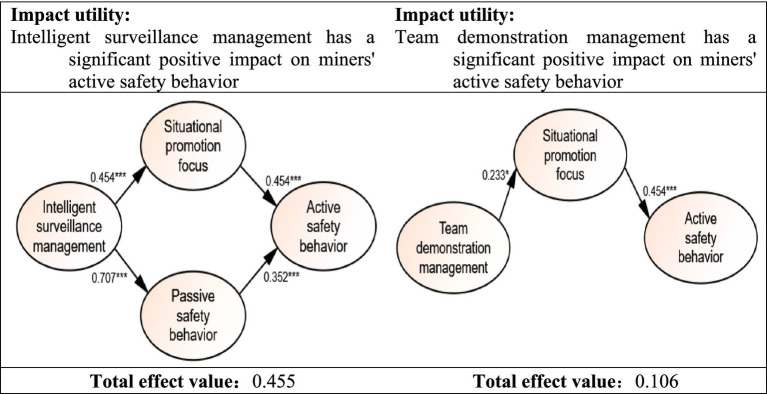
Comparison chart of path analysis results of significant influence effect.

Impact effect analysis encompasses three key aspects of effect analysis: the total impact effect, the direct impact effect, and the indirect impact effect (45). The direct impact effect is the effect value that the path coefficient can directly reflect; The indirect impact effect is a variable that indirectly affects the dependent variable by directly or indirectly influencing other variables, and its value is the product of the coefficients of two pathways; The total influence effect is the summation of the direct influence effect and the indirect influence effect, and the numerical value of the total influence effect is also referred to as the total effect value (47). The influence of intelligent surveillance management on coal miners’ active safety behavior is depicted on the left side of Figure 5. Meanwhile, the effect of team demonstration management on coal miners’ active safety behavior, along with its utility and total effect values, is presented on the right side of Figure 4. Overall, the effectiveness of intelligent surveillance management in terms of its impact on coal miners’ active safety behavior is 4.292 times greater than that of team demonstration management.

Adopting the conventional notation where *β* denotes the standardized path coefficients, the utility of Intelligent surveillance management is delineated by the following hierarchy of coefficients, *β_EIMF1_* exceeds *β_EIMP2_*, which in turn surpasses *β_EIMP3_*, followed by *β_EIMP1_*, and then *β_EIMF4_* equals *β_EIMF2_*, both of which are greater than *β_EIMF5_* and *β_EIMF3_*. Consequently, the sequence of utility exerted by the subindicators on miners’ safety behavior under the umbrella of Intelligent surveillance management is as such: Early warning of potential risks holds the greatest influence, succeeded by full-time risk prediction, risk decision support, real-time risk monitoring, duty compliance monitoring, standardized operation, facilitate the establishment of safety awareness and the development of safety skills with emergency assistance management. These elements are pivotal in shaping the outcome of the miners’ safety behavior.

### 4.2 Results analysis

Based on the analysis results of the structural equation model, it is evident that intelligent surveillance management exhibits a superior effect compared to team demonstration management in influencing miners’ safety behavior.

Firstly, intelligent surveillance management boasts a notable advantage in its dual intermediary regulation effect. Unlike team demonstration management, which solely relies on the situational promotion focus as an intermediary to impact miners’ active safety behavior, iIntelligent surveillance management leverages both the situational promotion focus and the situational prevention focus as mediators. The reason why intelligent surveillance management can exert its influence through these two intermediaries is its ability to not only identify scenarios conducive to safety behavior (situational promotion focus) but also predict and identify situations that may pose safety risks (situational prevention focus). This dual regulation mechanism enhances the flexibility and effectiveness of intelligent surveillance management in promoting miners’ active safety behavior.

Secondly, intelligent surveillance management boasts significant advantages in terms of both its overall utility value and strong path utility value. Specifically, the utility value of intelligent surveillance management in promoting miners’ active safety behavior is 4.292 times higher than that of team demonstration management. Furthermore, its utility value in enhancing the situational promotion focus is 1.95 times greater than the latter. This superiority stems from the fact that intelligent surveillance management can more precisely identify scenarios that either promote or prevent miners’ active safety behavior, allowing for more prompt feedback and intervention. Additionally, it has the capability to dynamically adjust its intervention strategies in response to changes in miners’ behavior and the surrounding environment, thereby ensuring the effectiveness of its interventions.

Finally, intelligent surveillance management boasts remarkable advantages in terms of real-time monitoring feedback and full-time control prediction. Real-time monitoring feedback demonstrates that intelligent surveillance management employs cutting-edge sensors, monitoring equipment, and data analysis technology to accurately monitor various parameters of the coal mine working environment and workers’ operational behavior in real-time. This real-time capability ensures that the system can respond promptly, reminding workers or automatically taking corrective measures whenever a potential safety risk or illegal operation is detected. On the other hand, full-time control prediction highlights the ability of the Intelligent surveillance and management system to continuously monitor the working environment and workers’ operations, providing detailed data and reports. This continuity and traceability not only facilitates timely identification and correction of safety issues but also serves as a solid foundation for follow-up safety training and improvement. In contrast, team demonstration management primarily relies on manual observation, demonstration, and oral guidance, which falls short in comparison to the real-time capabilities, continuity, and cost-effectiveness offered by intelligent surveillance management.

However, it is noteworthy that intelligent surveillance management does not exert a significant impact on miners’ passive safety behavior through either situational prevention focus or situational promotion focus. According to the investigation, miners have a limited recognition of the ability of intelligent surveillance management to enforce labor responsibilities and standardize operations. During their work, some workers demonstrate a certain level of awareness and responsiveness toward the deployment and control positions of intelligent monitoring equipment. Specifically, when these workers violate safety regulations, such as removing safety protection equipment, they intentionally avoid or block the intelligent monitoring equipment to evade surveillance. This behavior reflects their perception of the limitations of the intelligent monitoring system, assuming it can only monitor behavior in fixed positions and lacks effective enforcement of safety regulations. Nevertheless, this behavior not only exposes the potential shortcomings resulting from the incomplete coverage in the practical application of Intelligent surveillance but also highlights the psychological and behavioral conflicts miners may encounter. Consequently, future research should delve deeper into optimizing the design and implementation of the Intelligent surveillance and management system to enhance its effectiveness and acceptability in practical settings. Simultaneously, emphasis should be placed on enhancing miners’ sense of identity and compliance with safety regulations through comprehensive educational and training programs. This, in turn, will ultimately facilitate the efficient utilization of intelligent surveillance and management systems.

## 5 Discussion

By constructing a structural equation model, this paper verifies the significant effect of Intelligent surveillance management on improving coal miners’ safety behaviors and reveals its potential in investment strategies and future development. Innovatively, the research compares intelligent surveillance with traditional team demonstration management, finding that the former has a more comprehensive positive impact on miners’ proactive safety behaviors through dual mediation mechanisms: situational promotion and prevention focus. The total safety utility value of Intelligent surveillance management is 4.292 times higher than that of team demonstration management, providing a solid theoretical and practical basis for its popularization. Additionally, considering China’s unique mine conditions, the research emphasizes the importance of real-time monitoring and continuous prediction functions, proposing a management strategy that combines incentives and punishments to promote miners’ active participation. These findings are of great guiding significance for coal-mining enterprises and policymakers, aiding in optimizing resource allocation, improving safety management efficiency, and providing a scientific basis for strategy formulation.

The innovation of this research lies in applying the “regulation-situation-behavior” theoretical framework to the field of coal miners’ safety management in the digital and intelligent era (46), and conducting an in-depth analysis of the impact of digital intelligent technology on miners’ safety behaviors through a structural equation model. The research not only reveals the positive influence of Intelligent surveillance management on miners’ proactive safety behaviors through the dual mediation mechanisms of situational promotion focus and situational prevention focus, but also quantitatively compares the utility values of Intelligent surveillance management with traditional management methods, finding that the utility of Intelligent surveillance management is significantly higher than that of traditional methods. Furthermore, addressing the unique working conditions of deep mines in China, the research emphasizes the importance of real-time monitoring and continuous prediction functions, and innovatively proposes a comprehensive management strategy combining incentives and penalties to promote miners’ active participation and appropriate responses. These innovations not only provide a new theoretical perspective for research on coal mine safety behaviors but also offer practical management strategies for coal mine enterprises and policymakers, which is of great significance for promoting the sustainable development of the coal mining industry.

Although the study has achieved certain results, there are still some limitations for future research. For instance, the applicability and effectiveness of intelligent surveillance management may vary across different types of coal mining enterprises, which warrants further investigation in future studies. Additionally, the long-term effects and sustainability of intelligent surveillance management are issues of concern that merit in-depth exploration in subsequent research.

Future research should strive to expand the sample size and increase the sample diversity so as to improve the universality and extrapolation of the research results and ensure the heterogeneity of the research groups. The research needs to deepen and refine the variable dimensions of the situational promotion focus and the situational prevention focus, and conduct long - term follow - up research to evaluate the long - lasting effects of Intelligent surveillance management measures and their adaptability at different stages. In addition, miners’ perception of the limitations of the digital - intelligent system and the resulting reactive behaviors are worthy of in - depth study. It is necessary to explore miners’ psychological and behavioral reactions in the intelligent surveillance environment, identify and solve potential safety hazards, enhance miners’ sense of identity with the intelligent surveillance system and their willingness to abide by safety regulations, ensure the long - term effective operation and efficient utilization of miners and auxiliary digital - intelligent machines, provide theoretical and practical support for the in - depth application of intelligent digital technology in the coal mining industry, and promote the healthy and sustainable development of the industry.

## Data Availability

The raw data supporting the conclusions of this article will be made available by the authors, without undue reservation.
